# Deep Learning for Automated Prediction of Sphenoid Sinus Pneumatization in Computed Tomography

**DOI:** 10.2174/0115734056363158250429101521

**Published:** 2025-05-22

**Authors:** Ali Alamer, Omar Salim, Fawaz Alharbi, Fahd Alsaleem, Afnan Almuqbil, Khaled Alhassoon, Fahad Alsunaydih

**Affiliations:** 1 Department of Radiology, College of Medicine, Qassim University, Buraydah 52571, Saudi Arabia; 2 Department of Electrical and Computer Systems Engineering, Monash University, Clayton, VIC 3800, Melbourne, Australia; 3 Department of Electrical Engineering, College of Engineering, Qassim University, Buraydah 52571, Saudi Arabia; 4 Department of Computer Science, College of Computer, Qassim University, Buraydah 51452, Saudi Arabia

**Keywords:** Artificial intelligence, Deep learning, Convolutional neural networks, Sinonasal imaging, Computed tomography, Paranasal sinuses, Sphenoid sinus, Pneumatization

## Abstract

**Background:**

The sphenoid sinus is an important access point for trans-sphenoidal surgeries, but variations in its pneumatization may complicate surgical safety. Deep learning can be used to identify these anatomical variations.

**Methods:**

We developed a convolutional neural network (CNN) model for the automated prediction of sphenoid sinus pneumatization patterns in computed tomography (CT) scans. This model was tested on mid-sagittal CT images. Two radiologists labeled all CT images into four pneumatization patterns: Conchal (type I), presellar (type II), sellar (type III), and postsellar (type IV). We then augmented the training set to address the limited size and imbalanced nature of the data.

**Results:**

The initial dataset included 249 CT images, divided into training (*n* = 174) and test (*n* = 75) datasets. The training dataset was augmented to 378 images. Following augmentation, the overall diagnostic accuracy of the model improved from 76.71% to 84%, with an area under the curve (AUC) of 0.84, indicating very good diagnostic performance. Subgroup analysis showed excellent results for type IV, with the highest AUC of 0.93, perfect sensitivity (100%), and an F1-score of 0.94. The model also performed robustly for type I, achieving an accuracy of 97.33% and high specificity (99%). These metrics highlight the model's potential for reliable clinical application.

**Conclusion:**

The proposed CNN model demonstrates very good diagnostic accuracy in identifying various sphenoid sinus pneumatization patterns, particularly excelling in type IV, which is crucial for endoscopic sinus surgery due to its higher risk of surgical complications. By assisting radiologists and surgeons, this model enhances the safety of transsphenoidal surgery, highlighting its value, novelty, and applicability in clinical settings.

## 1 INTRODUCTION

The sphenoid sinus is an important surgical access point for many skull base lesions [1]. However, the variability of its pneumatization can complicate transsphenoidal surgeries, increasing the risk of injury to nearby neurovascular structures [2-4]. These structures include the internal carotid artery, optic nerve, cavernous sinus, and several cranial nerves [5-7]. Greater sinus pneumatization increases the prominence of these structures, putting them at greater risk of injury [8]; for example, one study on 3061 transsphenoidal surgeries reported 24 cases of vascular complications and 7 deaths [9], while another study on 1658 patients reported 11 cases of orbital complications, including 2 cases of optic nerve injury [10]. Non-pneumatized sphenoid sinuses were once considered a contraindication for transsphenoidal surgeries. However, advancements in the field now enable these surgeries to be safely performed using intraoperative image-guided drilling through the non-pneumatized sphenoid, provided the surgeon is informed in advance [11]. Evidently, identifying pneumatization patterns in computed tomography (CT) scans is crucial for surgical planning [12]; however, such findings are frequently missing from radiology reports.

**Fig. (1):**
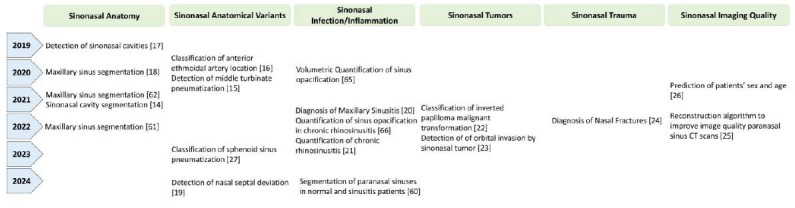
Application of DL in sinonasal imaging: Relevant key studies and their clinical contributions.

A deep learning (DL) model that automatically distinguishes various sphenoid sinus pneumatization patterns will be a useful tool for radiologists and surgeons alike. A review of the literature identified several studies that employed DL techniques for analyzing and interpreting radiological images of the sinonasal region (Fig. **1**). According to a recent scoping review from July 2023, DL models have been used on sinonasal images to segment anatomical structures and classify diseases such as sinusitis and tumors [13]. The imaging modalities used in these studies included X-rays, CT scans, and magnetic resonance imaging (MRI). Convolutional neural networks (CNNs) were utilized for the anatomical segmentation of various sinonasal structures, including the sinus lumen, nasal septum, turbinates, and anterior ethmoidal artery [14-19]. Researchers have also utilized DL on X-ray and CT scan images for the detection and classification of sinusitis, mostly in the maxillary sinuses [20, 21]. Lim *et al.* developed an auxiliary classifier-based multi-view network that can accurately predict the severity of maxillary sinusitis on X-rays and guide clinical decisions without the use of CT scans [20]. A semi-supervised and automatic segmentation algorithm that combines MobileNet, squeeze-and-excitation networks (SENet), and Residual Neural Network (ResNet) has also been used for three-dimensional (3D) volumetric CT scoring of chronic rhinosinusitis [21]. The DL classification capability was also investigated in sinonasal tumors, such as inverted papilloma, and in predicting the local invasion of sinonasal malignancies into the adjacent orbital region [22, 23]. One study has addressed the application of a 3D ResNet-based single-classification network for sinonasal trauma, focusing on the automated diagnosis of nasal fractures [24]. DL applications in sinonasal imaging extended beyond anatomical segmentation and disease classification to encompass quality improvement. One study evaluated the image quality of low-dose paranasal sinus CT imaging using a DL-based image reconstruction algorithm, as opposed to conventional reconstruction algorithms, and an improvement in overall image quality was observed [25]. Another study demonstrated an excellent performance of CNN models adopting ResNet-152 and DenseNet-169 architectures for the prediction of patients’ sex and age groups based on paranasal sinus X-rays, which can help to minimize the risk of patient misidentification [26]. The findings of these proof-of-concept studies indicate that DL-based approaches could have valuable applications in other aspects of sinonasal imaging, including the prediction of sphenoid sinus pneumatization patterns.

While several studies have explored the use of DL in sinonasal imaging, as outlined above, there is only one study in English literature that has investigated DL for the classification of sphenoid sinus pneumatization [27]. Taylor *et al.* developed a CNN model using the Microsoft Azure Custom Vision DL platform. They retrained the classification layer using the transfer learning method to tune the model for interpreting the sagittal CT images. The model achieved an overall accuracy of 85.9%. They used Hamberger’s classification system, which categorizes the pneumatization of sphenoid sinuses into three types: Conchal, presellar, and sellar [28]. However, there is an alternative classification system, presented by Güldner et al., which categorizes pneumatization into four types: Conchal, presellar, sellar, and postsellar [29]. The Güldner classification is likely more clinically useful for endoscopic transsphenoidal surgery, as it distinguishes between sellar and postsellar types, the latter of which is associated with a higher risk of surgical complications [3]. No prior studies have applied the Güldner classification for the development of a DL-based approach to distinguish the four pneumatization patterns.

Variability in sphenoid sinus pneumatization significantly impacts the safety of transsphenoidal surgeries. Therefore, the aim of our study was to develop an automated tool to assist radiologists and surgeons, ultimately improving patient care. The novelty of our approach lies in the use of CNNs to classify sphenoid sinus pneumatization according to Güldner’s classification system, which has not been previously applied in this context. This classification system is particularly relevant to the safety of endoscopic transsphenoidal surgery. An automated tool that makes use of this classification system will be a promising tool for preoperative surgical planning and reducing the risk of neurovascular injuries. We recognize the success and widespread use of CNNs in various image-related applications, leveraging their robust capabilities to process and analyze visual and spatial data [30, 31]. Additionally, the hierarchical structure of CNNs enhances classification performance and enables effective automatic feature extraction, minimizing the need for manual feature engineering and outperforming traditional machine learning methods [30, 32]. This is particularly relevant to our task, as the complexity and variability of sphenoid sinus anatomy can negatively impact the model's classification accuracy. Therefore, we opted for CNN-based algorithms for this medical imaging task. To address the limited size and imbalanced nature of the available dataset, we employed data augmentation and assessed its impact on the model’s performance.

## 2 MATERIALS AND METHODS

### 2.1 Data Collection, Dataset Preparation and Labeling, and Data Augmentation

This study (No.23-55-11) was approved by the Committee of Research Ethics, Deanship of Scientific Research, Qassim University, on September 14, 2023. In light of its retrospective design and the use of anonymous imaging data, the study was exempt from informed consent. Consecutive paranasal sinus CT scans performed between January 2023 and July 2023 were retrieved from a single-hospital Picture Archiving and Communication System (accessed on September 18, 2023). Patients with prior sinonasal surgery were excluded. No age limits were applied.

The CT examination was performed using the GE Revolution EVO 128 Slice scanner without an intravenous contrast medium. The source axial CT scan images were acquired in a caudocranial direction, spanning from the hard palate (inferiorly) to above the frontal sinuses (superiorly). The axial slice thickness was 0.625 mm, acquired at regular intervals of 0.625 mm, and encompassed a field of view ranging from 140 to 160 mm. The tube voltage and tube current were 120 kilovolts (kV) and 80–160 milliamperes (mA), respectively. The advanced adaptive statistical iterative reconstruction technique known as ASiR-V was employed to reduce the radiation dose (fixed noise index of 7.59 and 30% ASiR reconstruction). Additionally, coronal and sagittal images were acquired *via* multi-planar reconstruction.

Midline sagittal bone-window CT images of the sphenoid sinus were anonymized and extracted in TIFF format with an average resolution of 664 × 690 pixels. Two certified radiologists (A.A. and F.A.) with over 10 years of experience in their field assessed each image and classified the sphenoid sinus pneumatization into one of four patterns based on the Güldner classification [29]. The two radiologists were knowledgeable about the Güldner classification and collaboratively analyzed each image to reach a shared consensus. To classify a sphenoid sinus in terms of one of the four pneumatization patterns, the posterior wall of the sphenoid sinus is compared in relation to the anterior and/or posterior wall of the sella turcica on a mid-sagittal CT image [29]. Type I (conchal type) is characterized by the complete absence or only minimal pneumatization of the sphenoid sinus. In type II (presellar type), the posterior wall of the sphenoid sinus is positioned anterior to the anterior wall of the sella turcica. In type III (sellar type), the posterior wall of the sphenoid sinus is located between the anterior and posterior walls of the sella turcica. Finally, in type IV (postsellar type), the posterior wall of the sphenoid sinus is situated posterior to the posterior wall of the sella turcica. The appearance of these four patterns on mid-sagittal CT images is shown in Fig. (**2**).

The two-dimensional (2D) mid-sagittal image of the sphenoid sinus was deemed sufficient for the prediction of the four pneumatization patterns. For the purpose of standardization across patients and imaging techniques, the plane of interest was defined as the midline sagittal image that is perpendicular to the vomer and captures the most posterior aspect of the sphenoid sinus. Each input image incorporates different air-filled cavities, including nasal chambers, ethmoid air cells, frontal sinus, and sphenoid sinus, in addition to the air column in the oral cavity and pharynx. The sphenoid sinus, which is the focus of this study, represents the region of interest (ROI). We extracted the ROI of each image and converted it into 2D arrays defined as *R(M, N)*, where *M* and *N* represent the dimensions of the ROI array. The values of *M* and *N* are determined based on the ROI that covers the sphenoid sinus across all input images. After investigating several *M* and *N* values, we found that an *M* value of 130 and an *N* value of 220 would result in the most suitable ROIs. A binary mask is used to define the ROI of all the images, where mask pixel values of 1 indicate image pixels that belong to the ROI, while mask pixel values of 0 indicate image pixels that belong to the background. The binary mask images are obtained through a segmentation process. The segmentation was performed manually to identify and isolate the ROI within the images. This process ensured the creation of accurate binary masks, which were subsequently used as input for training the CNN. The binary mask is applied to the *R*(*M*, *N*) array as follows:

**Stage 1:** The maximum value within the array *R*(*M*, *N*), denoted as *max*(*R*(*M*, *N*)), is subtracted from each element in the array (eq. **1**):


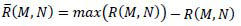


**Stage 2:** Each element of the resultant array in (1)-namely, 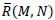-is divided by the maximum value within 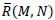, denoted by *max*(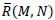) (eq. **2**):


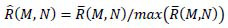


**Stage 3:** Each element of the resultant array in (2)-namely, 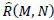-is compared with a threshold value (denoted by *η*) to indicate the background image pixels (eq. **3**):


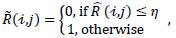


where 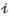 and *j* are the row and column indicators of the array 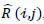, respectively, with 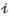 = 1, 2, . . ., *M* and *j* = 1, 2, . . ., *N*. As shown in Fig. (**3**), the ROI image pixels take values of 1, while the background image pixels take values of 0. The value of threshold *η* is determined by ensuring that all elements within the ROI are equal to one. After investigating several *η* values, we determined that an *η* value of 0.9 is optimal for achieving this objective.

The dataset was divided into training/validation and test sets using a random splitting method. The training/validation set comprised 70% of the data, while the test set was composed of the remaining 30%. When selecting the splitting ratio, we accounted for certain factors to ensure a balance between having sufficient training data and retaining enough data for a reliable evaluation. A 70/30 split was deemed to be optimal as, although the obtained dataset was limited and imbalanced, we intended to augment the training dataset. Augmenting 70% of the dataset would ensure that the model includes sufficient images for robust training, allowing more images (30%) to be allocated to the test dataset and facilitating a more reliable evaluation of the model’s performance metrics. Due to imbalances in the dataset, we applied the random splitting method to each class separately (stratified sampling) to ensure that the training and test datasets maintained the same class distribution.

We encountered two issues in our experiments: limited data and imbalanced classes. The number of images in one class (type IV) constituted more than half of the dataset, while the other types were minority classes. This is referred to as the imbalanced dataset problem. Therefore, we trained the CNN model on the original dataset and evaluated its performance, then employed data augmentation, trained the model on the augmented data, and evaluated its performance again for comparison. The data augmentation was employed for type I, II, and III pneumatization patterns in the training dataset in order to balance the data of these types with that of type IV, thus expanding the whole training dataset. We augmented these images through combinations of horizontal flipping, rotation (by 15°, 30°, 45°, 60°, 75°, or 90°), bounded translation, and bounding box cropping with upscaling. We opted for this combination of simple augmentation techniques to ensure balanced augmentation, achieving reasonable diversity while avoiding over-augmentation and unrealistic images. For example, we avoided using the shearing technique because it would produce unrealistic images. This is because the classification of the sphenoid sinus pneumatization pattern relies on the relationship between the pneumatized part and the anterior and posterior walls of the sella turcica, and when the shear transformation is applied to an image, the reference lines will be slanted, potentially leading to misclassification.

We chose the K-fold cross-validation technique, as it is a popular and effective method for evaluating machine learning models and was deemed suitable for our limited dataset. As we utilized data augmentation to balance the classes of the training dataset, class imbalance and biased performance estimates posed no issues when employing the K-fold cross-validation technique. When deciding whether to use 5-fold versus 10-fold cross-validation, we found the 5-fold cross-validation technique to be a better option for our data. This is because, if we used the 10-fold cross-validation technique, the training datasets per fold would be smaller, reducing the reliability of the training and increasing the chance of over-fitting. On the other hand, the 5-fold cross-validation technique would result in larger training datasets per training fold, facilitating more reliable training, a reduced training time, and a good balance between bias and variance.

### 2.2 CNN Model Development, Training, and Testing

**Fig. (4):**
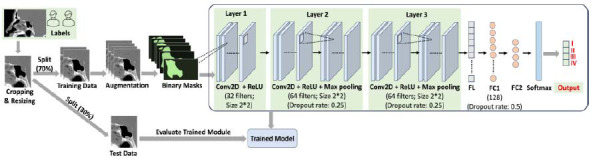
An overview of the data pre-processing and the proposed CNN model architecture. The model was trained on the training dataset before and after augmentation of the minority classes (types I, II, and III pneumatization patterns). Conv2D = convolutional layers; ReLU = rectified linear unit; Max pooling = maximum pooling layers; FL = flatten layer; FCL = fully connected layer.

The CNN model proposed for classifying sphenoid sinus pneumatization patterns (Fig. **4**) was developed using Python (version 3.11.1) and TensorFlow (version 2.14.0). The algorithm includes convolutional layers (Conv2D), maximum pooling layers, and rectified linear unit (ReLU) layers. These layers are connected to a flatten layer (FL) and fully connected layers (FCLs), the output of which is connected to a softmax layer. To optimize the performance of the CNN model, a random search was used for hyperparameter tuning. This method was selected due to its ability to efficiently explore a large hyperparameter space without the computational demands of a grid search. During the tuning process, key hyperparameters such as the learning rate, batch size, number of convolutional layers, filter sizes, and dropout rates were explored. The final selected parameters were a learning rate of 0.001, a batch size of 16, an epoch number of 10, and three convolutional layers with 32, 64, and 64 filters, respectively, with dropout rates of 0.25. The FCL had 128 neurons with a dropout rate of 0.5 and a ReLU activation function. The output layer was defined using the FCL with a number of neurons equal to 4 (reflecting the number of classes), and a softmax activation function was applied to convert the output into a cross-class probability distribution, making it suitable for multi-class classification tasks. An epoch number of 10 was chosen due to the size of the dataset, which contained only 249 samples. When the amount of data is limited, the risk of over-fitting increases significantly, as the model may quickly memorize the training data rather than learn to generalize well to new, unseen data. In such cases, training for too many epochs can lead to poor generalization, in which the model performs well on the training dataset but struggles with test data. Thus, the model parameters were selected based on their ability to achieve the best balance between accuracy and generalization, minimizing over-fitting while maintaining a strong performance on the validation set. In the Conv2D layer, filters with a size of (2, 2) were used to convolve the elements of the input array with the filter coefficients. The resultant values (**Y**) were passed to the next layer as follows (eq. **4**):


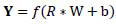


where *f* denotes the activation function, **R** represents the input, and **W** and **b** denote the weights and biases of the network, respectively, which are updated during the backpropagation step of network training. During the training process, the loss function, *η*, is calculated based on the probabilities of different classes (denoted by *K*) at the output of the softmax layer to evaluate the progress of training (eq. **5**):


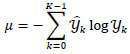


where 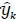 for *k* = 0, 1, . . ., *k* − 1 is calculated as (eq. **6**)


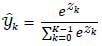


and *Z_k_* is the *k*^th^ element within the input vector *Z* = [*Z*_0_, *Z*_1_, . . ., *Z_K_*_−1_]^T^ to the softmax layer, 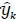 is the *k*^th^ element of the ground truth vector of 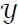, which can be expressed as 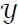 = [1, 0, . . ., 0*_K_*_−1_]^T^ for Class 0, as 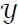 = [0, 1, . . ., 0*_K_*_−1_]^T^ for Class 1, and so on. The first step in training a CNN model is typically to set zeroes as the initial values of the biases (**b**) and random values for the weights (**W**). The ReLU layers are employed to eliminate negative values, such as ReLU(*x*) = *max*(0, *x*). The maximum pooling layers are employed to down-sample the feature map, thus reducing its spatial size and eliminating redundant spatial information. The maximum pooling layers in this study employed a 2 × 2 filter and a stride of 2 to choose the maximum value.

We calculated various model performance metrics before and after augmentation to determine the benefits of the process. These performance metrics included the area under the curve (AUC), accuracy, sensitivity (recall), specificity, positive predictive value (PPV), negative predictive value (NPV), and F1-score.

## 3 RESULTS

A total of 249 mid-sagittal CT scan images of the sphenoid sinus from 249 patients were included in this study. The age of patients ranged from 6 to 71 years (mean = 28, standard deviation = 13.89), including 135 (54.22%) male and 114 (45.78%) female patients. There was no statistically significant difference between both genders in this study (*p* = 0.183). Sphenoid pneumatization patterns of types I, II, III, and IV constituted 9 (3.61%), 28 (11.24%), 78 (31.33%), and 134 (53.82%) of the images, respectively (Table **1**). Of the 249 CT scan images, 174 (69.88%) images were used to train the developed CNN algorithm, while the remaining 75 (30.12%) were used to test the trained CNN algorithm. Table **1** outlines the distribution of the four patterns of sphenoid sinus pneumatization across the training and test datasets. The distribution of cases in these datasets mirrors that of the total dataset, with type IV being the most prevalent, followed by type III, type II, and finally type I, which has the lowest prevalence.

**Fig. (5):**
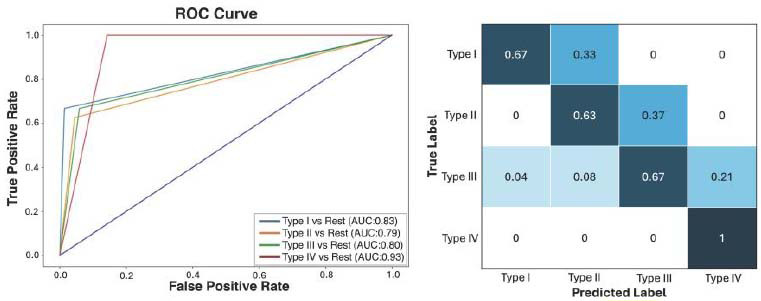
The receiver operating characteristic (ROC) curve, area under the curve (AUC), and confusion matrix showcase the performance of the trained CNN algorithm in predicting the four patterns of sphenoid sinus pneumatization.

**Fig. (6):**
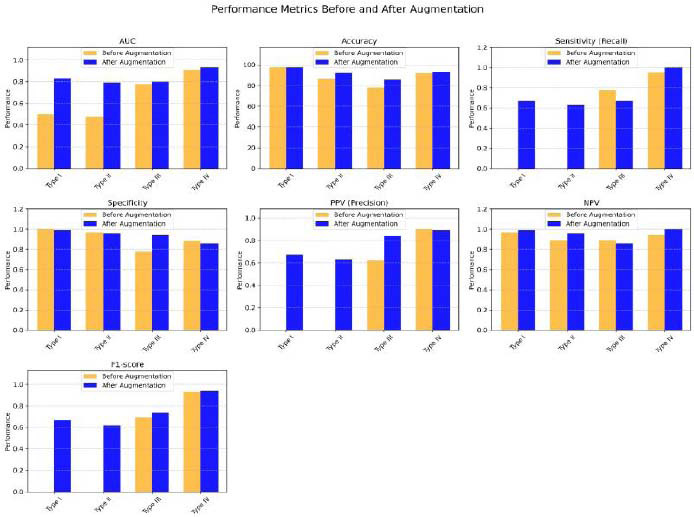
Performance metrics of the proposed CNN model before and after data augmentation.

Prior to data augmentation, the overall diagnostic accuracy of the proposed model was 76.71%, which represented an AUC of 0.67 (95% CI: 0.583–0.757), reflecting satisfactory diagnostic accuracy. After augmentation of the minority classes in the training dataset (from 174 to 378 labels), the model’s performance improved, with an overall diagnostic accuracy of 84% and an AUC of 0.84 (95% CI: 0.776–0.904), indicating very good diagnostic accuracy. The individual AUCs for the four pneumatization patterns significantly improved after data augmentation, with values ranging from 0.79 to 0.93 (Fig. **5**). Moreover, the other performance metrics, including sensitivity, specificity, PPV, NPV, and F1-score, also showed remarkable improvement following data augmentation, as illustrated in (Table **2** and Fig. **6**). Subgroup analysis revealed superior performance for type IV, evidenced by an AUC of 0.93, an accuracy of 93.33%, perfect sensitivity of 1, and an F1 score of 0.94. The notable boost in accuracy resulting from the data augmentation approach came at the cost of an increase in training time, from 186 seconds to 265 seconds.

## 4 DISCUSSION

Overwhelmed by their increasing workloads, radiologists may overlook findings, forget to report them, or make other kinds of mistakes due to simple human error [33, 34]. Artificial intelligence (AI)-based technologies can assist radiologists across various clinical applications, enhancing their efficiency, optimizing workflow, and improving the quality of patient care [35]. These benefits can be achieved throughout the patient’s imaging journey. For example, AI tools can aid in the protocol creation, worklist prioritization, image denoising, lesion detection, and semi-automated structured reporting [36, 37], potentially improving the turnaround times and enhancing the completeness of radiology reports [38]. However, transparency in AI-driven solutions is essential for building trust among clinicians and healthcare providers. It ensures that the decision-making processes of AI systems are understandable and accountable, thereby addressing concerns related to bias and ethical implications [39].

In this study, we present a CNN model that can automatically predict different patterns of sphenoid sinus pneumatization with very good diagnostic performance (AUC of 0.84). The model achieved an excellent performance (AUC of 0.93) in identifying type IV sphenoid sinus pneumatization, which is associated with a higher risk of neurovascular injury during transsphenoidal surgery. Prior studies have demonstrated the surgical importance of identifying the extent of sphenoid pneumatization. As the pneumatization of the sphenoid sinus expands-as is the case for type IV-there is an increased likelihood of adjacent vital neurovascular structures protruding into the sinus cavity [3, 8], increasing the risk of injury during transsphenoidal surgery. A previous study revealed that a large proportion (85%) of type IV pneumatization pattern was associated with protrusion of nearly all neurovascular structures, while only 31% of type III pneumatization patterns exhibited such protrusion [3]. Another study reported incidence rates of 0%, 0%, 7.32%, and 22.11% for optic nerve dehiscence in types I, II, III, and IV, respectively [8]. This study also showed incidence rates of 0%, 0%, 3.66%, and 13.16% for carotid artery dehiscence in types I, II, III, and IV, respectively. Another study highlighted the significant correlation between type IV and insertion of the intersphenoid septum into the internal carotid artery in 55.04% of cases, as opposed to 0% in types I and II [40]. If an avulsion fracture of the septum occurs during surgery, there is a risk of internal carotid artery injury and significant intraoperative hemorrhage [40, 41]. The thin bone covering the hyper-pneumatized sinus in type IV may also increase the risk of iatrogenic injury to the middle and posterior cranial fossae, resulting in parenchymal contusion, vascular injury, or cerebrospinal fluid leakage [3, 11, 40, 42]. Therefore, the CNN model proposed in this study has the potential to improve the safety of transsphenoidal surgery. It may assist radiologists in reporting paranasal sinus CT scans and help surgeons with pre-operative surgical planning, minimizing the risk of neurovascular injuries.

We encountered two problems that resulted in our CNN model underperforming after the initial training period; however, once these issues had been rectified, the model improved substantially, achieving very good to excellent performance. These problems included limited and imbalanced datasets, which are well-known and common challenges in healthcare applications of DL. With limited data, the trained model may achieve successful prediction of labels for the training data, but then perform poorly on new test data, demonstrating over-fitting and poor generalization [43-45]. This suggests that the limited data may lack diversity or comprehensiveness, hindering the model’s ability to effectively categorize various patterns of sphenoid sinus pneumatization found in different populations or imaging settings. Several techniques, such as data augmentation, transfer learning, dropconnect (*i.e*., randomly dropping connections between neurons), and batch normalization, can be implemented to avoid over-fitting and improve the performance of DL models [45]. Data augmentation is a commonly used approach in which the training set is expanded using techniques such as image rotation, flipping, translation, zooming, cropping, noise injection, contrast and brightness modifications, synthetic data augmentation, oversampling (*e.g*., based on the use of generative adversarial networks), and neural-style transfer augmentation [43, 45, 46]. When datasets are imbalanced, one class (the majority class) is significantly larger than the other classes (the minority classes). In this case, the DL model predicts the majority class with greater accuracy but performs poorly on the minority class, and may even ignore the minority class, treating it as noise. Moreover, using accuracy as a performance metric in such scenarios can be misleading [47]. This is because when the model correctly classifies all the majority labels but misclassifies all the minority labels, the resultant model accuracy will remain high as the number of majority labels surpasses the number of minority labels. The ROC curve and AUC are more reliable metrics in these instances. Several solutions can be used to tackle issues associated with imbalanced data [31, 48]. Such solutions can be applied at the data level, where datasets can be balanced by oversampling the minority class, under-sampling the majority class, or both (hybrid sampling) [45, 48-50]. Other solutions aim to optimize the model algorithms (algorithmic-level techniques), and can be combined with data-level techniques to form hybrid approaches, such as the class-boundary alignment algorithm, cost-sensitive learning, the label- and value-based weight algorithm, and ensemble techniques [48, 50, 51]. For example, Morais *et al.* employed novel algorithmic-level techniques, combining cross-entropy loss function with the sample-weighting approach using a learning optimal sample-weights strategy [52]. In their approach, they did not augment the small imbalanced datasets; instead, they used the available datasets and focused on the training phase. They optimized the model’s learning through estimating each training sample weight at several steps and determining which samples had the greatest contribution during the training process, forcing the model to learn to classify under-represented classes and preventing over-fitting to the dominant classes. On the other hand, Beddiar *et al.* worked to expand the training dataset through image augmentation [53]. They generated new images using a novel denoising CNN that denoised the original images, injected noise into the original images, and denoised the newly created images, resulting in three new sets of images. Due to the limited and imbalanced nature of the obtained data, our CNN model performed poorly on the minority classes (types I, II, and III), with AUC values of 0.50, 0.48, and 0.78, respectively. We augmented the training dataset for types I, II, and III in order to expand the overall training dataset, eliminate the data imbalance problem, enhance model training, rectify over-fitting, and improve the values of the performance metrics. After augmentation, notable improvements in the AUC metric were observed for types I, II, and III, yielding values of 0.83, 0.79, and 0.80, respectively. The sensitivity (recall), PPV, F1-score, and accuracy values also improved (Table **2**). Data augmentation not only resolves the issues of limited and imbalanced data, but also enhances the accuracy and generalizability of the model, improving its robustness across diverse populations and imaging settings [54]. The higher performance metrics for type IV were attributed to the availability of a larger sample of CT scans, constituting over half of the obtained dataset (53.82%). Therefore, increasing the amount of training data will likely result in further improvement of the diagnostic performance of our CNN model for the other subgroups. The uneven distribution of various patterns of pneumatization of the sphenoid sinus is well-documented in the literature. As was the case in our study, the conchal pattern showed the lowest prevalence (0%–8.5%) in other studies [3, 8, 11, 29, 41, 42, 55-57], and the reported ranges of the presellar pattern varied from 1.2%–27.2% [3, 8, 11, 29, 40-42, 55-57]. On the other hand, the most prevalent was either the sellar (14.6%–58.7%) [3, 11, 29, 40, 41] or postsellar pattern (5.6%–83.5%) [8, 42, 55-57]. In this study, we attempted to alleviate the imbalance in the dataset through the use of CT images obtained from pediatric patients. It is well known that the sphenoid sinus lacks pneumatization at birth [58]. Pneumatization becomes noticeable in the conchal region as early as 6 months of age [59], and it then expands both in the posterolateral and inferior directions [11]. Finally, the sinus reaches its mature size by the age of 14 [58]. Therefore, incorporating pediatric patients into the dataset could augment the number of minority classes (*i.e*., types I–III).

To date, only one study has explored the use of DL for the classification of sphenoid sinus pneumatization [27]. Their model classified the sphenoid sinus into three types, while our model classified it into four types. The models achieved comparable accuracy (85.9% and 84%). However, when attempting to identify the postsellar pneumatization pattern, which increases the risk of neurovascular injury during transsphenoidal surgery, our model demonstrated excellent diagnostic performance (AUC of 0.93), compared to the very good diagnostic performance (AUC of 0.86) achieved in the prior study. Our CNN model achieved an overall misclassification rate of 16%, which is comparable to the 17% misclassification rate achieved by Taylor *et al.* [27]. Luminal opacification of the sphenoid sinus can lead to misclassification, potentially incorrectly predicting an opacified sellar/postsellar sinus as a conchal or presellar sinus [27]. Using soft-tissue-window images instead of bone-window images could mitigate this issue. Whangbo *et al.* conducted a DL-based multi-class segmentation of the paranasal sinuses and found that performance metrics were negatively affected in opacified sinuses [60]. Increasing the size of the training dataset will likely reduce the misclassification rate, as opacification of the sphenoid sinus is uncommon, and having a larger dataset will reduce the impact of opacified sinuses. Another potential solution is 3D sinus segmentation using 3D CNN models, as prior studies have shown their excellent performance in separating bony borders of the sinus from its content-whether that content be gas or soft tissue opacification [61, 62]. Recently described feature fusion strategies have the potential to enhance model robustness by integrating multi-scale features to distinguish between overlapping structures [63, 64]. Applying these approaches to sphenoid sinus analysis could improve the reliability of pneumatization classification, especially in cases with sinus opacification.

A few other studies have explored other potential applications of DL in the context of imaging the sphenoid sinus. For example, one study employed semi-supervised DL semantic segmentation for 3D volumetric analysis of the sphenoid sinus in CT scans, achieving high (98.5%) accuracy [21]. The proposed method, which combines MobileNet, SENet, and ResNet, demonstrated enhanced segmentation accuracy compared to other state-of-the-art networks, such as Deeplab-V3+, which inadequately interpreted the boundaries of the sphenoid sinus, resulting in hazy output. These researchers also found that the sphenoid and frontal sinuses had a significantly higher asymmetric sidal difference than the other sinuses, contributing to the known anatomical variability of the sphenoid sinus. Other studies have applied CNN-based models to sinus CT scans for automatic segmentation and volumetric quantifications of luminal opacification of the paranasal sinuses, including the sphenoid sinuses [65, 66]. They confirmed the feasibility of CNNs in providing objective metrics that are comparable to CT-based visual scoring for chronic rhinosinusitis (Lund–Mackay scoring). Another study using MRI for neuronavigation of craniopharyngiomas employed a DL model for the segmentation of multiple surgically relevant structures, including the sphenoid sinus [67]. This model yielded a Dice coefficient of 0.877 for the localization of sphenoid sinuses on sagittal T1-enhanced MRI. Feng *et al.* used a CNN model to assess pituitary adenoma invasion into the sellar floor and reported a misclassified case due to type I sphenoid sinus pneumatization. However, this finding is not generalizable because of the small sample size [68].

This study has several limitations. We used a single 2D mid-sagittal CT image of the sphenoid sinus which was, in most cases, sufficient for proper identification of the pneumatization pattern. However, some patients exhibit variations in the sphenoid septum and asymmetry in the volume of the two paired sphenoid sinuses; thus, it can be challenging to rely solely on a single 2D mid-sagittal CT image for the assessment of sphenoid pneumatization, and the diagnostic accuracy may be affected. Therefore, our approach falls short of capturing the comprehensive 3D assessment that a skilled radiologist would undertake when examining a scan. Nevertheless, the degree to which utilizing 3D images may improve the performance of the CNN remains uncertain in the absence of prior comparative studies on the sphenoid sinus pneumatization. Extrapolating results from previous studies on vertebral fractures indicated that both a CNN model using 3D images [69] and another one using 2D images [70] yielded similar diagnostic performance results, with AUC values of 0.95 and 0.92, respectively. Thus, we may infer that there may be no major benefit of using 3D images for this specific task. A small dataset is another limitation, yet the proposed CNN was on par with other image classification algorithms employed in healthcare settings [16, 21, 62]. A post-hoc power analysis was conducted, utilizing the hypothesis, sample size, and proportional difference of the study. With an alpha value of 0.05, the power achieved was 99.72%, indicating that the sample size was sufficient to obtain statistically significant findings. Combining external datasets from multiple centers augments the sample size and validates the model's domain adaptation against inconsistent datasets across different populations, scanners, and imaging techniques [54]. However, inter-center heterogeneity hinders this approach. To address the challenges posed by heterogeneous datasets, an augmented multicenter graph convolutional network (AM-GCN) has been proposed [71, 72]. The reliance on manually derived binary masks can be time-intensive. Huang *et al.* propose a framework that enhances the interactive segmentation process by utilizing a click-based interaction method [73]. This allows users to provide input through simple clicks instead of engaging in extensive manual segmentation. The framework incorporates an iterative weighted loss function, enabling the model to effectively learn from user inputs. This significantly improves segmentation accuracy while reducing the time and effort needed for manual intervention.

## CONCLUSION

In this study, we developed a CNN model capable of identifying the extent of pneumatization in the sphenoid sinus and relating it to the sella turcica landmarks. The model demonstrated very good diagnostic performance in differentiating the four patterns of sphenoid sinus pneumatization. It was particularly outstanding for identifying type IV, which poses a higher risk of surgical complications in transsphenoidal skull base surgeries. Additionally, the model showed robust performance for type I, which is an important variant that surgeons need to know about in advance for better preoperative planning.

We employed simple image augmentation techniques that produce realistic images closely resembling real-life images. This approach significantly improved the model’s performance. We avoided augmentation techniques that generate unrealistic images, as they may improve performance on test datasets but lack generalizability, leading to potential failures when applied to actual clinical scenarios. The strong performance of this CNN model underscores its potential for reliable clinical application, making it a promising tool for assisting radiologists and surgeons.

Radiologists, often overwhelmed by increasing workloads, may overlook or forget to report such findings. This algorithm can help ensure the completeness of their reports, thereby improving the quality of care provided to patients. Similarly, this tool will assist surgeons in preoperative planning by alerting them to variants that require special equipment for drilling non-pneumatized sphenoid sinuses or that necessitate extra attention to nearby neurovascular structures at risk of injury in cases of extensive pneumatization. Thus, this model serves as a promising AI assistant for enhancing the safety of endoscopic transsphenoidal surgeries, highlighting its value, novelty, and applicability in clinical settings.

While our model demonstrated promising results, several limitations warrant further investigation. External validation is essential to objectively assess clinical efficacy and generalizability across diverse populations and imaging settings. Addressing inter-center heterogeneity may require AM-GCNs. Incorporating 3D sphenoid sinus imaging could improve pneumatization pattern prediction and surgical planning accuracy. Exploring advanced DL architectures, such as ResNet or DenseNet, may enhance feature extraction for complex medical imaging patterns. Transfer learning with pre-trained models (*e.g*., VGGNet, EfficientNet, or Inception) could also boost performance on smaller domain-specific datasets. Additionally, replacing manual segmentation with automated methods would improve efficiency and reliability in medical image analysis. Finally, ensuring transparency in AI-driven solutions is critical to mitigate bias and uphold ethical standards.

## Figures and Tables

**Fig. (2):**
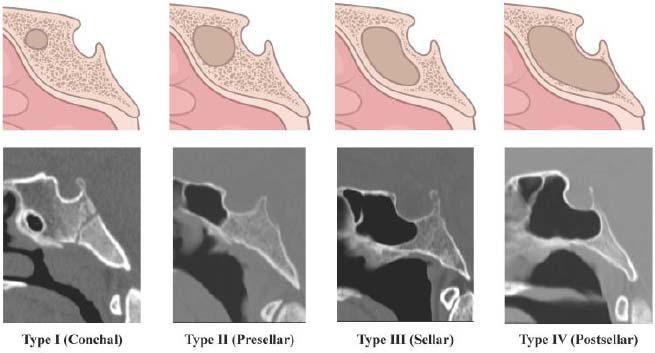
The diagrams (top row) and the corresponding mid-sagittal CT images (lower row) show the four pneumatization patterns of the sphenoid sinus (the illustrations in the top row were created using www.Biorender.com).

**Fig. (3):**
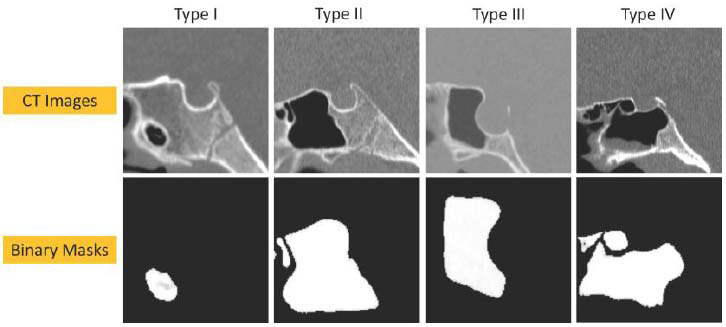
CT scan images (upper row) and corresponding binary masks (lower row) for the four pneumatization patterns of the sphenoid sinus.

**Table 1:** Distribution of the four patterns of sphenoid sinus pneumatization across the training and test datasets.

**Sphenoid sinus pneumatization pattern**	**Total** ***n* = 249**	**Training** ***n* = 174 (69.88%)**	**Test** ***n* = 75 (30.12%)**
Type I	9 (3.61%)	6 (3.45%)	3 (4%)
Type II	28 (11.24%)	20 (11.49%)	8 (10.67%)
Type III	78 (31.33%)	54 (31.03%)	24 (32%)
Type IV	134 (53.82%)	94 (54.02%)	40 (53.33%)

**Table 2:** Performance metrics of the proposed CNN model before and after data augmentation. AUC = area under the curve.

**Sphenoid sinus pneumatization pattern**	**AUC**	**Accuracy**	**Sensitivity** **(Recall)**	**Specificity**	**PPV** **(Precision)**	**NPV**	**F1-score**
**Type I**	0.83 (0.50)	97.33% (97.26%)	0.67 (0.00)	0.99 (1.00)	0.67 (0.00)	0.99 (0.97)	0.67 (0.00)
**Type II**	0.79 (0.48)	92.00% (86.30%)	0.63 (0.00)	0.96 (0.97)	0.63 (0.00)	0.96 (0.89)	0.62 (0.00)
**Type III**	0.80 (0.78)	85.33% (78.08%)	0.67 (0.78)	0.94 (0.78)	0.84 (0.62)	0.86 (0.89)	0.74 (0.69)
**Type IV**	0.93 (0.91)	93.33% (91.78%)	1.00 (0.95)	0.86 (0.88)	0.89 (0.90)	1.00 (0.94)	0.94 (0.93)

PPV = positive predictive value. NPV = negative predictive value. (Please note that the values in parentheses represent the model's performance before data augmentation).

## Data Availability

All data generated or analysed during this study are included in this article.

## LIST OF ABBREVIATIONS

CT: Computed tomography
DL: Deep learning
MRI: Magnetic resonance imaging
CNN: Convolutional neural network
SENet: Squeeze-and-excitation network
ResNet: Residual Neural Network
DenseNet: Dense Network
3D: Three-dimensional
2D: Two-dimensional
ROI: Region of interest
Conv2D: Convolutional layer
ReLU: Rectified linear unit
FL: Flatten layer
FCL: Fully connected layer
AUC: Area under the curve
ROC: Receiver operating characteristic
PPV: Positive predictive value
NPV: Negative predictive value
AI: Artificial intelligence
AM-GCN: Augmented multicenter graph convolutional network

## STANDARDS OF REPORTING

STROBE guidelines were followed.
